# A Case of Chronic Myeloid Leukemia Presenting as Bilateral Retinopathy

**DOI:** 10.7759/cureus.31718

**Published:** 2022-11-21

**Authors:** Varsha Kandambeth, Pranaykumar Shinde, Sachin Daigavane

**Affiliations:** 1 Department of Ophthalmology, Jawaharlal Nehru Medical College, Datta Meghe Institute of Medical Sciences, Wardha, IND

**Keywords:** exudates, fusion protein, hemorrhages, retinopathy, chronic myeloid leukemia

## Abstract

Chronic myeloid leukemia (CML) is a myeloproliferative neoplasm with raised granulocyte cell line. It arises in the hematopoietic stem cell and is characterized by the presence of BCR-ABL fusion gene as a result of translocation between chromosomes t(9,22) (q34; q11.2). CML affects bone marrow and peripheral blood. CML can be asymptomatic and is usually discovered on routine blood investigations. It can also present as symptoms associated with anemia and splenomegaly. Rarely do CML patients present with retinal abnormalities as an initial presentation. We cover a case of CML in this report that had retinal involvement as one of its earliest manifestations. Patient presented with gradual loss of vision in both eyes. Multiple bilateral intraretinal and preretinal hemorrhages with exudates were found during fundus examination. Blood tests revealed a raised leukocyte count along with lower hemoglobin levels. Peripheral smear revealed 4% blast cells. Bone marrow aspiration showed hypercellular marrow, marked granulocyte proliferation, and raised myeloid erythroid ratio. The diagnosis was further confirmed by fluorescence in-situ hybridization (FISH) which showed BCR-ABL1 fusion gene. Patient was immediately started on chemotherapy and has been on follow-up since then. Visual acuity of the patient improved and there was no progression of retinopathy. This case thus serves as an illustration of how early detection and management can significantly slow the progression of retinopathy and improve visual outcomes.

## Introduction

Chronic myeloid leukemia (CML) is a myeloproliferative neoplasm with raised granulocyte cell line. It arises in the hematopoietic stem cell and is characterized by the presence of BCR-ABL fusion gene diagnosed by routine cytogenetics or BCR-ABL1 abnormalities by fluorescence in situ hybridization (FISH) or by molecular studies [1,2].

CML patients may experience ophthalmologic symptoms. They are either a direct outcome of leukemic cell infiltration or in some instances, ocular symptoms are a result of secondary indirect causes, such as hematologic abnormalities brought on by leukemia, central nervous system involvement, opportunistic infections, and severe drug reactions. Leukemia has typically been identified in individuals before they visit an ophthalmologist. However, in certain cases, CML can present with ocular symptoms which may be discovered on detailed examination and investigations. Thus timely management and diagnosis help the patient to get a favorable visual outcome and better disease prognosis.

## Case presentation

**Figure 1:**
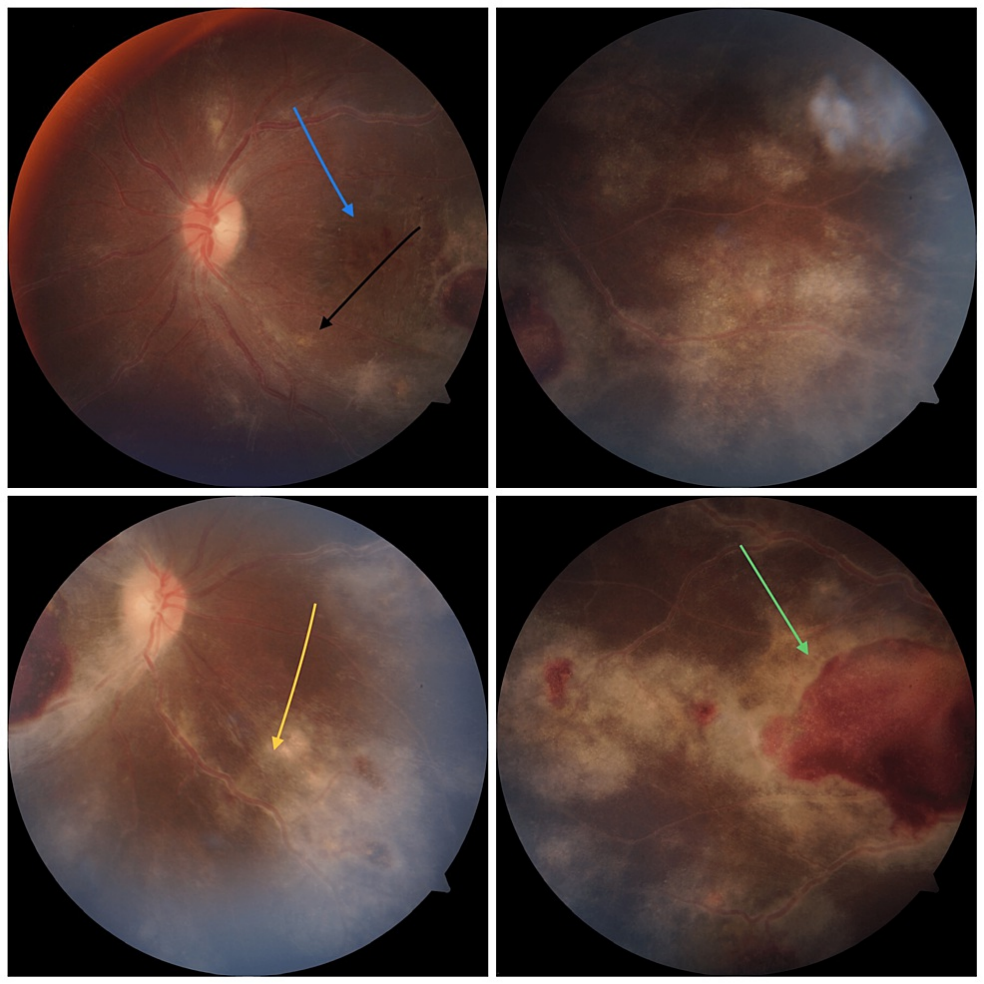
Fundus photo showing clear media with pale disc in right eye. Intra-retinal and pre-retinal hemorrhages are shown with the help of blue and green arrows, respectively, the yellow arrow shows perivascular sheathing and the black arrow shows cotton wool spot.

Patient is a 31-year-old female, homemaker by occupation from central India. She came to the department of ophthalmology outpatient unit at a tertiary care hospital around one year ago with complaints of painless, progressive loss of vision of six months duration, in right eye more than left. It was also associated with occasional headache which was throbbing in nature. There is no history of associated redness, trauma, hypertension, or diabetes mellitus. No history of intake of any eye medications or systemic medications in the past. There was no significant family history. On examination, the patient had best corrected visual acuity of 6/60 in right eye and 6/12 in left eye. There was grade 2 relative afferent pupillary defect (RAPD) in right eye, intra-ocular pressure measured by applanation tonometry was within 18 mmHg in both eyes. Dilated fundus examination showed arterial attenuation, tortuous vessels, perivascular sheathing, and intra-retinal and pre-retinal hemorrhages with cotton wool spots in all quadrants in both eyes along with pale disc in right eye (Figure 1).

The patient was advised blood investigations. Blood investigations showed leukocytosis with reduced hemoglobin count, coagulation profile was within normal limits (Tables 1, 2). Chest x-ray, ultrasound abdomen, and ECG (electrocardiogram) showed no abnormalities. Peripheral smear showed myeloblast 1%, promyelocyte 8%, myelocyte 20%, metamyelocyte 10%, neutrophil and band forms 43%, eosinophils 9%, basophil 7%, monocyte 1%, and lymphocyte 1%. Bone marrow aspiration showed hypercellular marrow, myeloid erythroid ratio 6.1:1, erythroid cells 14%, myeloblast 4%, promyelocyte 3%, myelocyte 20%, metamyelocyte 5%, polymorphs and band forms 38%, basophils 5%, eosinophils 7%, lymphocytes 3%, and plasma cells 1% suggesting chronic phase of CML. Fluorescence in situ hybridization (FISH) further confirmed the diagnosis by detecting BCR-ABL1 fusion gene. Patient was immediately started on chemotherapy with imatinib. During her follow-up after one year, there was no progression in retinopathy or decline in visual acuity. Visual acuity in right eye was 6/60 and left eye improved to 6/9.

**Table 1:** Hemoglobin, platelet count, and renal function test.

Total WBC count	3,22,300/mL
Hemoglobin (%)	7.1 g/dL
Platelet	1.44/mL
Creatinine	0.9 mg/dL
Potassium	4.3 mmol/L
Sodium	141 mmol/L
Urea	15 mg/dL

**Table 2:** Coagulation profile of the patient.

Activated partial thromboplastin time (APTT) - control	APTT - patient	Prothrombin time - control	Prothrombin time - patient	INR
29.5 seconds	30.0 seconds	11.9 seconds	13.4 seconds	1.13

## Discussion

CML (chronic myeloid leukemia) is a myeloproliferative neoplasm with raised granulocyte cell line. It arises in the hematopoietic stem cell and is characterized by the presence of BCR-ABL fusion gene as a result of translocation between chromosomes t(9,22) (q34; q11.2). CML affects bone marrow and peripheral blood [3]. CML is a triphasic disease with chronic, accelerated, and blast phases, and symptoms may vary according to the phase. In chronic phase, patients may present with symptoms of anemia and splenomegaly. As the disease progresses to accelerated and blast phase symptoms, such as headaches, bone pain, joint pain, and lymphadenopathy are more common [4]. Ocular manifestations in initial phases are usually rare and have been described through only few case reports over time [5,6]. Similar retinal findings can be found in other retinopathies and hence might be missed initially, especially if the patient has other systemic disorders [7]. Hence it is important to evaluate the patient and advise baseline investigation before jumping to a diagnosis. In our case, the patient did not have significant improvement of vision in right eye due to optic nerve involvement. However left eye showed improvement in vision after starting the patient on chemotherapy with imatinib, above all, there was no further worsening of retinopathy. There have been a few reported cases of retinal involvement in acute stages of CML and many cases have shown improvement in visual acuity with treatment. Five-year survival rate was also significantly higher in patients who received treatment early [8]. Hence early detection and treatment are of prime importance in saving the patient's life and vision.

## Conclusions

Chronic myeloid leukemia presenting with retinopathy is rare and can be mistaken for other retinopathy if not investigated properly. Hence it is important to thoroughly investigate any patient with retinopathy for leukemia and treatment should be started early to save the life and eye of the patient.
